# Cysts of the Spermatic Cord and Testicle

**Published:** 1894-03

**Authors:** James E. Thompson

**Affiliations:** M. D., M. B., B. S., Lond. Univ., F. R. C. S., Eng.; Professor of Surgery, University of Texas; Galveston


### TEXAS MEDICAL JOURNAL.

ESTABLISHED JULY, 1885.

PUBLISHED MONTHLY.—SUBSCRIPTION $2.00 A YEAR.

VOL. IX. AUSTIN, MARCH, 1894. NO. 9.

### Original Contributions.

For Texas Medical Journal.

### CYSTS OF THE SPERMATIC CORD AND TESTICLE.

BY JAMES E. THOMPSON, M. B., B. S., LOND. UNIV., F. R. C. S., ENG.
Professor of Surgery, University of Texas.

CYSTS of the spermatic cord and testicle are usually the results of morbid processes occurring in embryonic structures. In the cord, the funicular process is responsible for most of the cysts. This process may close only at two points, viz.: just above the testicle and at the internal ring, leaving a fully formed tunica vaginalis and a cavity above, which may fill with fluid and form a long, sausage-like cyst reaching the whole length of the cord. Again, the process may be obliterated at intervals, leaving a string of potential cavities which, when filled with fluid, appear like beads on a string. In the lower part of the cord, just above the testicle, the cysts may have a different origin. We have here a portion of the Wolffian body, the so-called organ of Giraldès, remaining functionless, but whose ducts may at any time become distended with fluid, forming a cyst which can with difficulty be distinguished clinically from those lying in the head of the epididymis.

Cysts of the epididymis usually occupy the head of that organ, their most common situation being the upper angle where the head joins the testicle. Here they result from distension of the hydatid of Morgagni (upper end of Mullerian duct) with fluid, and the cyst projects into the cavity of the tunica vaginalis.

The other cysts in the head of the epididymis have one of two origins: (1) from one of the vasa efferentia, the contents often being a milky fluid full of spermatozoa (spermatocele), or (2) from an accumulation of fluid in the connective tissue lying between the convoluted tubules of the epididymis. Occasionally, but rarely, a cyst may be found lying between the visceral layer of the tunica vaginalis and the tunica albuginea (encysted hydrocele of the testicle). Another rare condition is a cystic dilatation of the vas aberrans (one of the posterior tubules of the Wolffian body), producing a tumor situated at the tail of the epididymis. The two last conditions are so rare that they need little consideration.

The diagnosis of these cysts is usually very easy, but occasionally it is quite impossible to distinguish clinically between the different varieties. Cysts of the cord, and particularly those which lie in the inguinal canal, are often mistaken for hernia. I have been often called in consultation on cases of this nature, only to find, on careful examination, that one had to deal with a hydrocele of the cord. Usually, by traction on the testicle, the cyst can be pulled out of the inguinal canal, and a careful examination will demonstrate its translucency. This symptom is not to be relied on altogether in young children, as an enterocele free from fæcal contents often transmits light perfectly. There is usually an impulse on coughing in these cases, and this becomes more marked the nearer the cyst lies to the internal ring. This impulse is not exactly like that given by a true hernia; it is less expansile and less direct. It requires experience to be fully appreciated. Cysts occupying a position lower down the cord are easily recognized; when associated, however, with an irreducible hernia, their signs may be masked. I operated once for radical cure of hernia, and removed three cysts of this nature from the anterior surface of the hernial sac.

The cysts of the testicle and those lying at the head of the epididymis can hardly be distinguished from one another with certainty, nor, before operation, is it of supreme importance that they should. One should, however, be careful not to mistake the thickenings of the epididymis resulting from gonorrhœa for a cyst of new formation.

Cysts due to the echinococcus are so rare that they hardly need special consideration.

The treatment of these conditions is essentially unsatisfactory, and often leads to much heartburning and discontent on the part of the patients. Recognizing that my earlier results in treating these cysts by puncture and injection of some antiseptic irritant had been attended by almost uniform failure, I have, during the last few years, whenever I have been permitted, attacked them by the open method, making a complete dissection and removing them entirely. This method I have employed not only in cases of cysts lying in the spermatic cord and the head of the epididymis, but have followed Bergmann's plan of treating ordinary hydrocele of the tunica vaginalis by complete excision of the parietal layer. Although this method may seem somewhat radical, I am convinced that in careful and skillful hands it is less innocuous than the treatment by injection. Given a clean, quick operator, and a well prepared patient, the case progresses from first to last without the slightest disturbance; pain is an exception, and suppuration an unknown quantity.

The preparation of the patient is perhaps the most important part of the whole procedure. It is an exceedingly difficult matter to thoroughly disinfect the scrotum, because the hair follicles, in spite of the most careful washing, still retain micro-organisms which may infect our wound. The scrotum and pubes should be carefully shaved the night before, and boracic acid fomentations applied for twelve or fourteen hours, until within two hours of the time fixed for operation. The scrotum is then washed again, and carefully wrapped in a towel soaked in bichloride of mercury, 1-1000. In this condition the patient is anæsthetized, and, when thoroughly unconscious, the part is again washed, shaved, and irrigated with bichloride. By this means we can in most cases disinfect the skin, but oftentimes we may find to our dismay that stitch abscess will result.

The conduct of the operation depends on whether the cyst is seated in the cord proper, or projects into the cavity of the tunica vaginalis. If in the cord, the cyst is pinched up between the left thumb and forefinger, and an incision made over it parallel to the direction of the cord. Occasionally this is hard to accomplish, particularly if the cyst be situated in the inguinal canal. However, in most cases, if an assistant drags on the testicle, the cyst can be drawn outside of or up to the orifice of the external ring, and an incision is made over it in this situation. I have never had occasion to slit up the aponeurosis of the external oblique to get at a cyst, but, should occasion require, I should do it without the slightest hesitation. Having cut carefully through the skin and the fascial coverings of the cord, the cysts can, as a rule, be easily shelled out, and, if multiple and arranged like beads on a rosary, they should be dissected out one by one. Great care should be taken not to injure the vas deferens during this performance, but no particular fear need be entertained if the spermatic veins are torn, although any injured vein must be carefully tied (not twisted) to prevent the wound from subsequently filling with blood. The veins of the cord have a great tendency to reserve their bleeding until the operation is finished, and a careful search should always be made before closing, to see that no small vessel has been left unsecured. It is often insisted that if the spermatic artery be wounded, there is great risk of sloughing or atrophy of the testicle. I have wounded and tied the spermatic artery dozens of times in the operation for varicocele, and have never found such a result. All that is necessary is to preserve the integrity of the vas deferens and its artery, and the testicle will remain intact.

Occasionally the cyst may be very hard to separate from the structures of the cord. In one case, after having determined to operate, I found when the patient was on the table that the cyst was exceedingly difficult to find. After a careful dissection the lower end was isolated, the vas deferens was with great difficulty separated from it, and a cyst fully one inch long was removed from the portion of the cord normally lying in the inguinal canal. It was not necessary here to slit up the external oblique muscle, as traction on the testicle enabled me to pull the cyst entirely outside the external ring.

If the cyst lie in the head of the epididymis, it will be necessary in almost all cases to open the cavity of the tunica vaginalis. An incision is made through the parietal layer large enough to allow the testicle to slide out, and the cyst will at once come into view. The visceral layer of the tunica vaginalis is carefully incised and separated from the cyst wall by the blade of a blunt pointed pair of scissors. A few side movements are sufficient to enucleate the cyst from its bed, and it can in most cases be removed whole. This method applies more to those cysts which result from dilatation of the hydatid of Morgagni (Mullerian duct) than to cysts which lie deeply embedded in the substance of the epididymis, and which result from dilatation of one or more vasa efferentia. These latter may become very large and numerous. In one case, I found that the epididymis was occupied by four large cysts which seemed to communicate with one another, and which had caused atrophy of the whole organ, necessitating castration. If the cyst can be completely removed, as above described, the rent in the visceral coat of the tunica vaginalis is united with a catgut suture, and the testicle replaced in the parietal coat, another catgut suture being placed in this. The treatment of the skin incision requires some care. It is my practice to unite the deeper tissues together with a few buried catgut sutures, and then to suture the edges of the skin separately. Halstead's suture is an excellent one for these wounds, but I have found that if care is not taken, the edges of the skin have a great tendency to turn in and prevent union by first intention. I therefore employ numerous interrupted sutures of catgut or horsehair; the catgut sutures having this advantage, that they need not be removed. The horsehair sutures are non-irritating, and can be left in for a week or ten days. A dressing of iodoform gauze is placed over the wound, and sublimated cotton over this. The penis is pulled through a hole in the dressing, and over all is placed a square of oiled silk, with an aperture left for the penis to emerge. The dressing is then fastened on with a triangular bandage, and strict orders are given that the patient is not to be removed from the recumbent posture for at least three days. It is as well, if the patient is in a private house, to look forward to the necessity of drawing off the urine for a short period after the operation. Retention of urine is often a troublesome complication, but seldom lasts longer than twenty-four hours.

